# Cell-type-specific memory consolidation driven by translational control

**DOI:** 10.1038/s41392-021-00471-0

**Published:** 2021-01-29

**Authors:** Qing Zhang, Isabel Bestard-Lorigados, Weihong Song

**Affiliations:** 1 Townsend Family Laboratories, Department of Psychiatry, The University of British Columbia, Vancouver, BC Canada; 2 Institute of Aging, School of Mental Health and Kangning Hospital, The Second Affiliated Hospital and Yuying Children’s Hospital, Wenzhou Medical University, Wenzhou, China

**Keywords:** Molecular neuroscience, Neurological disorders

Dr. Sonenberg’s group has significantly contributed to the understanding of translational control in learning and memory. In a recent *Nature* article, they further reported the cell-type-specific translational control underlying memory consolidation.^1^

Long-term potentiation (LTP) has been widely acknowledged as one of the main cellular models of learning and memory. Generally, there are two components of LTP driven by different stimulations and mechanisms. The early phase of LTP (E-LTP) and short-term memory (STM) are evoked for short periods and are temporary, relying primarily on modifications of pre-existing proteins. The late phase of LTP (L-LTP) and consequent long-term memory (LTM) are elicited by strong and repetitive stimulation and are long-lasting, requiring new protein synthesis.^2^ Lowering the threshold for LTP induction can facilitate the transition from E-LTP to L-LTP and lead to memory consolidation. Therefore, deciphering the specific molecular machinery driving L-LTP formation is essential for understanding learning and memory. Dr. Sonenberg’s group previously discovered that eukaryotic initiation factor 2 (eIF2), a master controller of protein synthesis, is one molecular switch between STM and LTM.^3^ In a recent issue of *Nature* titled “eIF2α controls memory consolidation via excitatory and somatostatin neurons”, the same group further elucidated the cell-type-specific mechanisms of memory consolidation driven by eIF2.^1^

eIF2α is the phosphorylatable component of the eIF2 ternary complex, which drives translation initiation. In higher eukaryotes, the eIF2α subunit can be phosphorylated by four kinases under integrated stress response (ISR), namely PERK, PKR, HRI, and GCN2 (Fig. 1a). Phosphorylated eIF2α (p-eIF2α) at Ser51 drastically inhibits general translation, while paradoxically upregulating a subset of mRNAs with upstream open reading frames such as *Atf4*. On the other hand, phosphatases such as the GADD34/PP1c and CReP/PP1c complexes can dephosphorylate eIF2α.^4^ Learning can reduce p-eIF2α levels, potentially via a mechanistic model involving kinase activity regulation, such as GCN2, the main eIF2 kinase in the brain (Fig. 1a). Research using GCN2^−/−^ mice showed increased gene transcription and enhanced L-LTP, leading to LTM after performing weak training tasks. During learning processes, by decreasing GCN2 and consequently reducing p-eIF2α, there might be less inhibition of CREB-dependent gene transcription, facilitating L-LTP and resulting in memory consolidation.^5^ In agreement with notions that L-LTP is dependent on new protein synthesis and that p-eIF2α suppresses translation, Dr. Sonenberg’s group further found that p-eIF2α impairs LTM while inhibition of p-eIF2α enhances LTM,^3^ arguing for eIF2α as a molecular switch for memory. However, mice with homozygous non-phosphorylatable *Eif2a* mutation Ser51Ala died within 1 day after birth.^3^ Therefore, it is essential to target p-eIF2α specifically to avoid undesirable side effects. In this recent *Nature* article, Sharma et al. revealed that eIF2α facilitates memory consolidation, particularly in excitatory neurons and somatostatin (SST) expressing inhibitory neurons.^1^

**Fig. 1:**
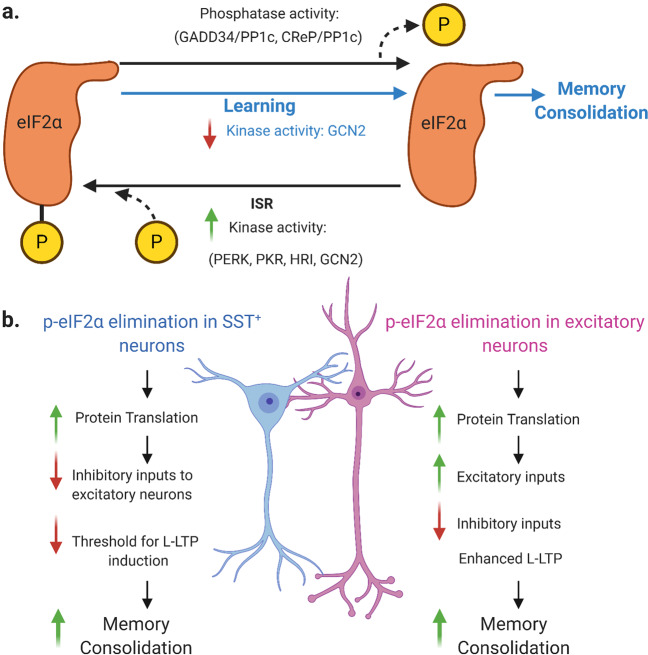
Role of eIF2α phosphorylation in memory consolidation. **a** PERK, PKR, HRI, and GCN2 kinases can phosphorylate eIF2α during integrated stress response (ISR); while phosphatase activity of GADD34/PP1c and CReP/PP1c complexes causes dephosphorylation. Learning can reduce eIF2α phosphorylation, leading to increased general protein translation and facilitation of memory consolidation. Modulation of kinase activity, such as decreasing GCN2 activity, is one proposed mechanism behind the learning-related reduction of p-eIF2α.^5^ **b** Elimination of phosphorylated eIF2α (p-eIF2α) in somatostatin (SST) neurons and excitatory neurons lead to memory consolidation via different autonomous pathways. In SST^+^ neurons, ablation of p-eIF2α results in increased general protein translation, fewer inhibitory signals to pyramidal neurons of the hippocampus, which can lower the threshold required for L-LTP induction leading finally to increased long-term memory. Ablation of p-eIF2α in excitatory neurons of the hippocampus also enhances protein translation, resulting in increased excitatory inputs and reduced inhibitory ones, which enhances L-LTP and leads to memory consolidation. Image created with Biorender.com

At the beginning of this article, the authors found that p-eIF2α is reduced by active learning in a cell-type-specific manner, occurring in excitatory neurons and SST-expressing inhibitory neurons. Therefore, they tested the hypothesis that eIF2α regulates memory consolidation through translational controls in excitatory and SST^+^ inhibitory neurons via cell-type-specific elimination of p-eIF2α. To achieve the effective elimination of p-eIF2α without lethality, this study used a transgenic mouse carrying homozygous non-phosphorylatable *Eif2a* mutant Ser51Ala and a wild-type *Eif2a* transgene flanked by *loxP* sites (*Eif2a*^*A/A*^*fTg*^*+*^). By crossing *Eif2a*^*A/A*^*fTg*^*+*^ mice with different Cre-expressing mice, p-eIF2α was selectively eliminated in excitatory neurons (in *Eif2a* cKI^*Camk2a*^ mice), inhibitory neurons (in *Eif2a* cKI^*Gad2*^ mice), SST^+^ inhibitory neurons (in *Eif2a* cKI^*Sst*^ mice), and parvalbumin (PVALB^+^) inhibitory neurons (in *Eif2a* cKI^*Pvalb*^ mice), respectively.

The authors first investigated the impact of p-eIF2α elimination on memory consolidation in excitatory neurons. While STM was not affected at the behavioral level, LTM was substantially enhanced in *Eif2a* cKI^*Camk2a*^ mice. A validation experiment injecting AAV9-Camk2a-Cre into hippocampi of *Eif2a*^*A/A*^*fTg*^*+*^ mice also enhanced LTM. Previous research has shown that p-eIF2α inhibits protein synthesis at the translational level.^4^ As expected, p-eIF2α elimination in excitatory neurons enhanced protein synthesis and resulted in a similar translational profile as learning processes. Protein synthesis is a prerequisite for L-LTP, the putative mechanism underlying LTM. In line with previous findings that p-eIF2α impairs L-LTP,^3^ p-eIF2α elimination in excitatory neurons enhanced excitatory inputs while reducing inhibitory inputs, facilitating the transition from E-LTP to L-LTP. Collectively, results from the above three aspects demonstrate that p-eIF2α elimination in excitatory neurons enhances protein translation underlying the induction of L-LTP, thereby facilitating the consolidation of LTM (Fig. 1b).

Using similar approaches, the authors further investigated the impact of p-eIF2α elimination in inhibitory neurons. They found that ablation of p-eIF2α in inhibitory neurons (in *Eif2a* cKI^*Gad2*^ mice) or SST^+^ inhibitory neurons (in *Eif2a* cKI^*Sst*^ mice) significantly facilitated memory consolidation. Furthermore, they revealed via electrophysiological experiments that p-eIF2α elimination in SST^+^ inhibitory neurons might promote memory consolidation by two mechanisms: disinhibition of excitatory neurons, thus facilitating LTP induction, and repression of inputs from the entorhinal cortex. Although p-eIF2α elimination in PVALB^+^ inhibitory neurons also enhanced general protein translation, it did not affect LTP or LTM.

Overall, this is the first study deciphering the cell-type-specific mechanisms of memory consolidation controlled by eIF2α phosphorylation. Enhanced general translation in excitatory neurons facilitates L-LTP expression, while changes in SST^+^ inhibitory neurons promote memory at the circuit level. These two types of neurons may work in harmony to facilitate memory consolidation (Fig. 1b). Furthermore, eIF2α phosphorylation occurs in various stress conditions and neurological disorders; therefore, understanding more about its cell-type-specific expression during learning and memory could offer clinical implications. For example, p-eIF2α and three stress-responsive kinases (PKR, PERK, GCN2) are activated in Alzheimer’s disease, owing to the unfolded protein response triggered by Aβ aggregates, and tau oligomers.^4^ The increased p-eIF2α could further exacerbate memory loss, forming a vicious cycle. Nevertheless, phosphorylation of eIF2 is a dynamic process that can be reversed by active learning (Fig. 1a). It implicates a positive loop of learning and memory: on the one hand, learning per se promotes the formation of LTM; on the other hand, mechanistically, learning decreases p-eIF2, thus facilitating the consolidation of LTM. As indicated in this study, cell-type-specific translational control by eIF2 phosphorylation is fundamental to memory consolidation.

## References

[1] Sharma V (2020). eIF2α controls memory consolidation via excitatory and somatostatin neurons. Nature.

[2] Kandel ER (2001). The molecular biology of memory storage: a dialogue between genes and synapses. Science.

[3] Costa-Mattioli M (2007). eIF2α phosphorylation bidirectionally regulates the switch from short- to long-term synaptic plasticity and memory. Cell.

[4] Moon SL, Sonenberg N, Parker R (2018). Neuronal regulation of eIF2α function in health and neurological disorders. Trends Mol. Med..

[5] Costa-Mattioli M (2005). Translational control of hippocampal synaptic plasticity and memory by the eIF2α kinase GCN2. Nature.

